# A Review of Technological Forecasting from the Perspective of Complex Systems

**DOI:** 10.3390/e24060787

**Published:** 2022-06-04

**Authors:** Lijie Feng, Qinghua Wang, Jinfeng Wang, Kuo-Yi Lin

**Affiliations:** 1 School of Management Engineering, Zhengzhou University, Zhengzhou 450001, China; ljfeng@shmtu.edu.cn (L.F.); 202012302014995@gs.zzu.edu.cn (Q.W.); 2 China Institute of FTZ Supply Chain, Shanghai Maritime University, Shanghai 201306, China; 3 School of Business, Guilin University of Electronic Technology, Guilin 541004, China; 21005005@guet.edu.cn

**Keywords:** technological forecasting, complex systems, data analysis, co-occurrence network, literature review

## Abstract

Technology forecasting (TF) is an important way to address technological innovation in fast-changing market environments and enhance the competitiveness of organizations in dynamic and complex environments. However, few studies have investigated the complex process problem of how to select the most appropriate forecasts for organizational characteristics. This paper attempts to fill this research gap by reviewing the TF literature based on a complex systems perspective. We first identify four contexts (technology opportunity identification, technology assessment, technology trend and evolutionary analysis, and others) involved in the systems of TF to indicate the research boundary of the system. Secondly, the four types of agents (field of analysis, object of analysis, data source, and approach) are explored to reveal the basic elements of the systems. Finally, the visualization of the interaction between multiple agents in full context and specific contexts is realized in the form of a network. The interaction relationship network illustrates how the subjects coordinate and cooperate to realize the TF context. Accordingly, we illustrate suggest five trends for future research: (1) refinement of the context; (2) optimization and expansion of the analysis field; (3) extension of the analysis object; (4) convergence and diversification of the data source; and (5) combination and optimization of the approach.

## 1. Introduction

Technology forecasting (TF) is the systematic study of scientific, technological, economic, and social developments in the longer term. Its goal is to identify strategic research areas and select the technology clusters that will make the greatest contributions to economic and social interests [1]. As an important stage in the process of technological innovation, TF can find the development trends of future technology from existing information [2]. Therefore, commercial organizations and government agencies widely adopt the systematic technology foresight method to embrace new technologies for competitiveness [3]. In recent years, many scholars have studied the methods and tools, application fields, and rationality of TF [4,5,6]. Some studies also focus on specific research areas, such as medical status and trend analysis [7], technology diffusion models in artificial intelligence [8], and technology integration in manufacturing [9]. Despite extensive research on TF, the issue of selecting the complex processes that best characterize the organization has been ignored in the literature [3].

No TF can ignore its complex interactive nature, and it cannot be evaluated without its context [10]. As far as the concept of TF is concerned, it refines the existing information with the help of the beforehand control principle of system cybernetics to predict the future developments in science and technology, such as trend extrapolation [3]. In terms of TF methods, the Delphi [11] method needs to consider not only the driving force of technology itself, but also other factors affecting technology development in the system [12]. TF is an important way to improve the national innovation system while providing enterprises and the public with a channel to understand and engage in technological development. Prediction not only studies the technology itself, but also needs to consider the interactions with other things. Therefore, this paper regards TF as a complex system, which is the result of positive and repeated interactions between individuals and other individuals in the system in a specific context.

A complex system is an organic whole composed of interconnected and interacting elements. It includes both the interactive and adaptive relationships between subject components and the synergistic evolutionary relationships between subject and relational components [13]. Differently from the traditional scientific research, complex system science can directly point to problems based on context theory and promote theoretical progress [14]. As research continues to advance, the study of complexity science has evolved from purely theoretical contributions to applications relevant to real-life. For example, complex systems provide a new perspective to explain and solve the complex and often counterintuitive behavior of social systems and their macro-level collective dynamics [15]. Meanwhile, the development of network science provides a tool to simplify the complex systems picture. While retaining the ability to see how the system is stitched together into a whole, we can gain insight into the operation of the system by exploring information from networks [16]. Thus, despite the complexity of TF, the idea of complex systems engineering can be integrated into the research of TF to promote its development. By reviewing studies from leading journals in technological innovation, this paper explores the context of TF, analyzes the agents covered within its system, and explores its dependencies. To achieve the research purpose, the following key research questions (RQs) were defined:

RQ1: What is the context in the complex system of TF?

RQ2: What are the agents in the complex system of TF?

RQ3: What is the interactive relationship between agents?

To address the research questions, we identify the contexts and agents and perform a social network analysis that can help us understand the system of TF comprehensively. Firstly, CiteSpace is used to systematically analyze research hotspots of TF and clarify system research boundaries, leading to capturing the context in complex systems of TF. Secondly, the main agents are identified with the help of the term frequency-inverse document frequency (TF-IDF) and Latent Dirichlet Allocation (LDA) topic models. Finally, social network analysis is utilized to correlate the contexts and agents for deeper exploration of correlations within the TF system.

The division of this article is as follows. Section 2 describes the research method and the process of literature review, and Section 3 introduces the results of TF. Section 4 discusses the impact on practice. Finally, Section 5 suggests the limitations and future research.

## 2. Methods

### 2.1. Search and Selection Process

**Figure 1:**
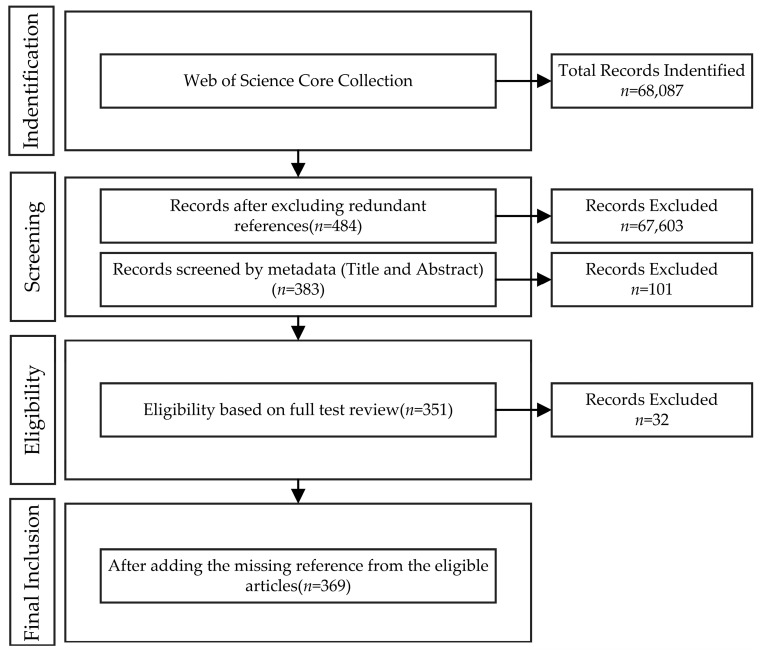
The search and selection process goes through identification, screening, eligibility, and final inclusion stages to obtain documents that meet the review criteria.

This paper uses a systematic literature review to achieve its research objectives and answer the research questions, because it is a comprehensive and systematic approach [17]. Systematic research can ensure the usefulness and feasibility of a review [18]. Therefore, this paper followed the Preferred Reporting Items for Systematic reviews and Meta-Analysis (PRISMA) to improve the quality of literature. Four stages are included in this flow chart: identification, screening, eligibility, and final inclusion. The data search and selection process is shown in Figure 1.

Identification Stage: This stage is the process of literature research and collection. Consistent with the related terms used in the previous search for relevant literature [19], we searched and retained 68,087 articles containing certain search terms in titles, keywords, and abstracts in the Web of Science (WoS) Core Library. The WoS was chosen because of its rigor and extensive literature that included peer reviews. The search and collection process was conducted on 24 November 2021.

Screening Stage: The screening process was divided into two phases. Firstly, following Thongpapanl, N [20], the top 50 journals in the field of technology and innovation management (TIM) were used to qualify articles for highly relevant literature. Amongst the 68,087 articles, 484 articles remained. Then, we used the following screening criteria to manually screen the titles, keywords, and abstracts in the remaining articles: (1) articles that were not a review of the literature; (2) articles that were not related to TF literature; (3) the article introduces at least one method of TF. There were 383 articles remaining after two stages of screening.

Eligibility Stage: In this stage, we read the full text of the above-remaining articles and retained the literature closely related to RQs. In this process, 351 articles met the requirements after excluding 32 articles.

Final Inclusion Stage: The purpose of this stage was to increase the effectiveness of research. By traversing the references of the above articles, 18 articles related to research issues were added. Therefore, 369 articles were finally retained for subsequent analysis.

### 2.2. Measures of the Study

To identify its context and agent and understand the complex structure of participation, we reviewed the literature by employing qualitative and quantitative analysis. Specifically, this included three aspects: (1) the acquisition of contexts in the system of TF; (2) the acquisition of agents in the system of TF; (3) the analysis of interactive relationships in the system of TF.

The research of context theory is problem-oriented, and the context of the system itself is the core variable for examining the behavior and evolution of the system. To systematically grasp and classify the context of TF, we used CiteSpace software to cluster the literature and divided the context of the system of TF into four categories. Firstly, the above 369 articles were imported into CiteSpace software, and the literature was clustered using the log-likelihood rate (LLR) algorithm. Secondly, the clustering topics were merged with synonyms to obtain more accurate contexts. Finally, a content analysis of the literature corresponding to each topic was performed to fine-tune the classification results of clustering topics.

The agent is the basic element of the complex system, which can promote the evolution of the whole system in continuous interaction [21]. To classify and understand different agents of TF, we constructed a morphological matrix and decomposed the system into four types of agents. Firstly, previous TF articles were reviewed to construct the initial version of the matrix. Secondly, LDA (article-theme matrix and topic-word matrix) and TF-IDF were used to further adjust the matrix. Identifying and classifying important keywords utilizing text mining could explore and discover potential agents, which can complement and improve the agent matrix of TF. Finally, the matrix was fine-tuned by manual reading.

Complexity is the result of the interaction between many agents [22]. To effectively explore the complex interactions between agents in the TF system, we constructed a complex network to explore the interaction between agents in different contexts. Firstly, based on the context and agent identified above, the full context-agent and single context-agent co-occurrence matrices were constructed. Secondly, the relationship was visualized with Gephi [23] software, which provides information not only about the network structure but also about the relationships of the network nodes. Finally, the research status and future research trends of the TF field are analyzed and explored.

### 2.3. Synthetic Analysis

The qualitative content analysis ran through the whole process of this analysis to make up for the limitations of quantitative analysis. Considering that there are many tools and methods involved in the TF system, we drew on existing research [4,24] and divided these models and methods into nine categories according to their purposes and mechanisms. (i.e., expert opinions, trend analysis, text analysis, statistical methods, modelling and simulation, network analysis, clustering, association, descriptive and matrices). This paper also analyzes the complex system in each context, and these phenomena show the interactions between agents in the TF system. The results are visualized by the network. In each network, nodes and links represent the contexts, agents, and their interactions. Specifically, nodes with different colors represent different agents. The size of a node represents the frequency of corresponding elements in the dataset. The strength of the interlinking lines represents the frequency of joint occurrence of nodes.

## 3. Results

This paper systematically analyzes TF-related articles from different knowledge sources and addresses three RQs presented in the introduction. The complexity of the interactions between TF agents is demonstrated through the above analysis, and how different agents apply these interactions is explained from a systems perspective.

### 3.1. What Is the Context in the Complex System of TF?

**Figure 2:**
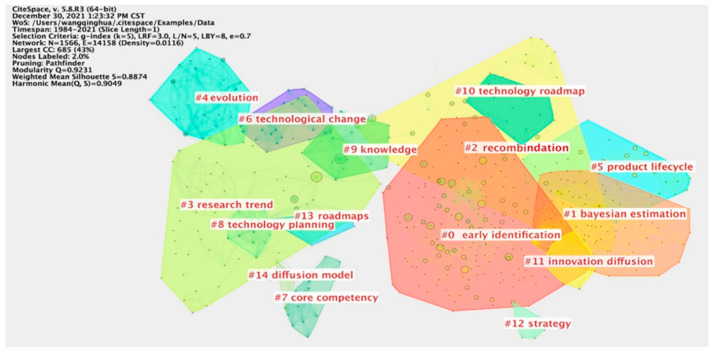
The figure shows 15 clustering topics in the system of TF, which can classify and reveal the contexts of TF. The network density was 0.0116, and the weighted mean silhouette was 0.8874, which indicates that the clustering results were reasonable.

To address RQ1, we used CiteSpace [25] software to conduct cluster analysis on TF-related articles data and obtained Figure 2. There were 1566 nodes and 14,158 lines in the TF sample literature network during the study period. The network density was 0.0116, and the weighted mean silhouette was 0.8874, indicating that the clustering contour was good, the clustering keywords were closely linked, and the clustering results were reasonable. Fifteen clustering topics were obtained based on keywords.

Some studies divide the context of TF into impact assessment, national foresight studies, roadmap, and competitive technological intelligence [26]. We further classify the above 15 topics based on reading the literature on clustering topics, as shown in Table 1. TF can be used in four contexts: (1) technology opportunity identification; (2) technology assessment; (3) technical trend and evolution analysis; and (4) others. Firstly, TF can be used to identify and explore emerging technologies [27,28], disruptive technologies [29,30], vacant technologies [31,32], and key technologies [33,34]. Secondly, TF can also emphasize technology to analyze the causes and influences of technology’s invention, innovation, and evolution in the whole system [35,36], and to evaluate the maturity or life cycle of technology [37,38]. Thirdly, TF can track the evolution of technology in a certain period [39,40]; show the internal relationship among the market, product, and technology in the form of the technology roadmap [41,42]; and study the diffusion mechanisms of technology [43,44]. Finally, the use of TF in technical cooperation and competition [45], knowledge management [34], and decision support is gradually increasing [46].

### 3.2. What Are the Agents in the Complex System of TF?

**Figure 3:**
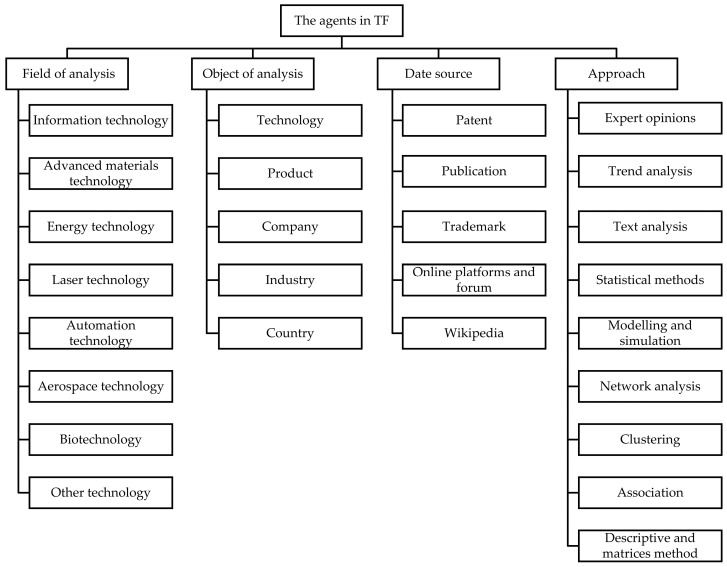
Summary of the literature review with the distinct elements for each agent.

To address RQ2, we systematically combed the literature on the TF by using quantitative analysis as the main method and qualitative analysis as the auxiliary method. It is considered that the system of TF covers four types of agents: (1) field of analysis; (2) object of analysis; (3) date source; and (4) approach. These four types of agents are interrelated and interact with each other, forming a complex system of TF. The slight changes of any agent in the system will lead to different forms of feedback iteration. Figure 3 shows four main types of agents in the systems of TF.

The system of TF consists of four types of agents: (1) field of analysis; (2) object of analysis; (3) date source; and (4) approach. These agents are the basic elements that contribute to the continuous evolution of the system.

#### 3.2.1. The Field of Analysis Involved in the Complex System of TF

**Figure 4:**
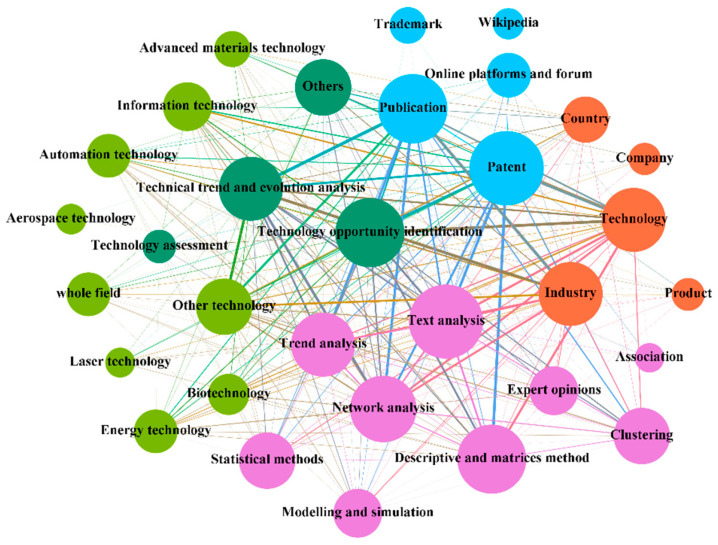
The figure shows the interactive relationship between the contexts and agents, which can provide deeper insights into the functioning of the system of TF.

The problems solved by TF are extensive and can be applied to many different fields. However, different applications have different technical characteristics, which profoundly affect the way we deal with forecasting and foresight. Combined with the specific meaning of the international patent classification number, the specific fields involved in TF are classified into the corresponding technical fields. TF now covers eight technical areas: (1) information technology; (2) advanced materials technology; (3) energy technology; (4) laser technology; (5) automation technology; (6) aerospace technology; (7) biotechnology; and (8) other technology. Among them, energy technology, information technology, automation technology, and biotechnology have received the most attention as application areas of TF (Figure 4). Previous studies of TF often focused on a single field, and there was a limitation of weak universality of prediction methods [27]. However, in recent years, it has gradually turned into multi-field research to explore the applicability, effectiveness, generality, precision, correctness, and robustness of the technology development model [58]. In addition, some studies carried out TF for the whole field [59]. Additionally, their forecasting methods can guide R&D subjects to explore their strengths and weaknesses [60], and the results of the studies can provide R&D subjects with visual insights into technology prospects [61]. Table 2 shows some concrete fields for each field in TF systems.

#### 3.2.2. The Object of Analysis Targeted in the Complex System of TF

Technology also plays an important role in shaping society as a whole [77]. Technological development is dependent not only on its internal logic but also on the conditions of the invention and the use of the object itself. Specifically, TF involves five types of analysis objects: (1) technology, (2) product, (3) company, (4) industry, and (5) country. Most studies draw upon a single patent database for technical analysis [27], or they make use of a database with patents and publications from various sources to identify technology opportunities [2]. Additionally, some studies are separated from a single technical analysis, and a product innovation method is selected depending on the economic and technical strength of the company [78]. Meanwhile, part of the research focuses on gathering and analyzing accurate, forward-looking, and actionable intelligence about the business environment, competitors, and the organization itself at the company level. This can help managers assess competitors and suppliers to improve competitive efficiency and effectiveness [63]. Finally, there is also a part of the study that analyzes industries [53] and countries [8]. Its data sources include patents incorporating technical information, trademarks with product information, and online forums with market information. Thus, the logical relationship between technology, industry, and country can be measured.

#### 3.2.3. The Data Source Employed in the Complex System of TF

TF uses existing knowledge and information to describe technology development patterns and minimize future uncertainty [79]. The choice of data sources affects the selection of TF methods and the accuracy of forecasting results. This paper identifies five types of data sources involved in TF: (1) patents; (2) publications; (3) trademarks; (4) online platforms and forums; and (5) Wikipedia.

Firstly, patents are the most frequent source of data and are considered a direct proxy for identifying the level of technology because they feature an up-to-date and reliable source of technical intelligence [80]. Patent databases such as the United States Patent and Trademark Office (USPTO) [81], Derwent Innovations Index (DII) [27], The European Patent Office (EPO) [41], and the Korean Intellectual Property Office (KIPO) [79] have been widely used to realize TF in numerous contexts.

Secondly, publications play an important role in accelerating and stimulating technological innovation as a major output of scientific activity [2]. Publications can be used to track and detect trends and evolution in research fields [82]. In addition, a combination of patent databases and publications can also be used to achieve technology opportunity identification by revealing the relationship between science and technology.

Thirdly, a trademark declares the ownership of products and services designated by the applicant. It covers information related to the applicant that can provide competitive intelligence. Additionally, products or services protected by trademarks can generate promotional advantages and technological advantages [32]. Trademarks can be used alongside patent databases to identify new business opportunities related to a company’s main business area [63].

Fourthly, online platforms and forums cover information that is the collective wisdom of multi-stakeholder participation and key information for vision formation [83]. Researchers and developers can obtain user needs from user review-based data to guide subsequent R&D innovation efforts [84]. They can also tap into future relevant expertise and insights on forum platforms based on the participatory comments of experts, researchers, and consultants who are interested in a particular field [51].

Fifthly, Wikipedia databases cover the globe and provide accurate and up-to-date information that can be used for a wide range of technology and innovation management research [85]. Wikipedia can be used to explore the composition of technologies and identify the connections between technologies [86]. In addition, a technological ecology network can be constructed based on Wikipedia, which in turn can monitor and analyze systematic changes in technology [9].

#### 3.2.4. The Approach Facilitated in the Complex System of TF

In technology forecasting and foresight activities, the choice of methods is closely related to the quality of forecasts. According to different contexts and technology analysis, TF requires many different methods to describe the future vision and grasp the development trends. According to the research results [4], this paper summarizes the research methods of TF, as shown in Table 3.

Firstly, expert opinions are obtained by consulting professionals through correspondence or on-site in-depth interviews to obtain forecasting results. In this group, the scenario analysis method is more widely used. It makes various scenarios or forecasts about the future development of the forecast object by showing the interactions of the development trends and critical times in various fields [67,87].

Secondly, the trend analysis method uses past data to make predictions. It is represented by the bibliometric approach [88] and regression curves [89]. Bibliometrics is a mathematical and statistical method for exploring the current state and trends of research. The most applied regression curve is the S-curve [90], which can be used to estimate when a specific stage in the innovation life cycle will take place.

Thirdly, text analysis methods quantify feature words extracted from text to represent textual information. Among them, Subject–Action–Object (SAO) analysis [66] mines technical features from a semantic perspective, which can eliminate subjective understanding bias to a certain extent. Additionally, LDA [91] uses technical topics to characterize thematic features. It has a stronger characterization ability compared to keyword analysis [34].

Fourthly, statistical methods rely on observed data to predict subsequent developments by establishing connections between them. The models used for prediction are usually quantitative in nature. Among statistical methods, correlation analysis and regression analysis [92] are often used. Parametric tests [9] are also frequently used to evaluate the predicted results.

Fifthly, modeling and simulation can use mathematical means to abstract complex problems into models that encompass all technical systems and help find which innovative research is most valuable. It can help clients find the best way to approach a problem, face a test, or solve a problem [47]. The accuracy of system simulations depends on the realism of the data and the way relationships are communicated in the simulation model.

Sixthly, network analysis can deal with complex problems with multiple relationships and help find the required information in the clutter of connected relationships. Citation networks explore the development of scientific fields and the relationships between disciplines by citing and being cited in the literature [68,93] and are also the most widely used network analysis methods.

Seventhly, the clustering method can group a collection of objects into multiple categories consisting of similar objects. It can be used as an independent tool to obtain the distribution of data, observe the characteristics of each cluster of data, and focus on a specific set of clusters for further analysis [94].

Eighthly, association analysis looks for frequent patterns, associations, correlations, or causal structures that exist between data. Association rules focus on correlations between technical characteristics [95], and causal analysis focuses on the causal feedback relationships between factors [87].

Ninthly, the descriptive matrix approach lies in interpreting valid information and expanding innovative ideas. Among them, the patent map [69] and knowledge map [96] reflect the intricate information. Additionally, the TRIZ method [64] explains the laws and principles inherent in innovation, and the morphological analysis (MA) [75] method searches for original and practical solutions through morphological analysis.

**Table 3:** The table shows the major models and methods for each approach involved in the system of TF and provides a brief description for each.

Approach	Model and Method	Description	Ref.
Expert opinions	Focus groups	This method observes the views and reactions of the respondents to something.	[97]
	Delphi	This method is a process of collective anonymous thought communication in the form of correspondence.	[98]
	Scenario planning	This method can make assumptions or projections for the future development of the forecast object.	[99]
Trend analysis	Bibliometrics	This method can explore the current situation and trends in the research field.	[40]
	Logistic curve	This approach shows the evolution pathway of the overall system of technology over time.	[58]
Text analysis	Keywords analysis	This method uses keywords or high-frequency words to represent the characteristics of the research field	[34]
	SAO analysis	This method extracts the Subject-Action-Object structure from the text and explores the characteristics of the research field from the semantic perspective	[73]
	LDA	This method explores topic distribution in text based on the Bayesian algorithm.	[81]
	Latent semantic analysis	This method excavates topic distribution in text based on singular value decomposition (SVD).	[86]
	Hidden Markov model	This method describes the process of generating random unobservable random sequences by Markov chain and then generating observable random sequences by each state.	[100]
Statistical methods	Sequential pattern mining	This method can mine patterns with high relative time or other patterns.	[92]
	Parametric test	This method uses sample data to infer the overall distribution pattern.	[9]
	Principal component analysis	This method reduces the dimension of original features by statistical methods.	[41]
Modeling and simulation	Agent model	This method uses the approximate model to simulate a high precision simulation model.	[101]
	Cross-impact analysis	This method considers the interaction and influence of technology and predicts based on finding vacancies.	[102]
	Genetic algorithm	This method can solve complex combinatorial optimization problems.	[103]
	Backtracking algorithm	This method is an optimal search method, according to the optimal conditions to search forward to achieve the goal.	[73]
	Neural network	This method is a mathematical model for distributed parallel information processing by imitating the behavior characteristics of animal neural networks.	[47]
Network analysis	Citation network	This methodology can reflect the history, context, and structure of the development of science and technology	[93]
	Co-citation network	This method reveals the content correlation and implicit co-occurrence relationship between keywords, classification numbers, authors, and other meaningful fields.	[104]
	Time-axis network	This method takes months and years as the axis to study the inheritance and development of technology	[44]
	Network-Based on Node Similarity	This method uses SAO semantic analysis, association rules, and other tools to mine the relationship between nodes to build a network.	[73]
Clustering	Hierarchy-based	This method creates a clustering tree and tree graph by calculating the similarity between nodes.	[105]
	Density-based	This method assumes that the clustering structure can be determined by the tightness of the sample distribution (e.g., DBSCAN algorithm).	[106]
	partition-based	This method enables you to partition a dataset into a specified number of clusters (e.g., K-means)	[94]
Association	Spatiotemporal association rule	This method can reflect the interdependence and relevance between one thing and others	[95]
	Causal analysis	This method uses the causal relationship between the development and change of things to predict.	[87]
Descriptive and matrices method	Patent map	This method organizes patent information into a variety of analytical chart information.	[69]
	Knowledge map	This method is a knowledge navigation system and shows important dynamic relationships between different knowledge stores.	[96]
	TRIZ	This method reveals the inherent laws and principles of the invention and obtains the final ideal solution based on contradictions.	[64]
	MA	This method is a sub-functional combination solution method for systematic search and stylized solutions.	[76]
	Multi-angle evaluation	This method uses different indicators to evaluate technology standardization from multiple perspectives.	[107]

### 3.3. What Is the Interactive Relationship between Agents?

To address RQ3, it is necessary to integrate content analysis methods based on network analysis to explore the matching relationship between specific contexts and each agent. Highly cited literature, as the core literature of the research field, can effectively identify the research component of the field [108]. Meanwhile, the literature from the last ten years can effectively represent the frontier of field research. Therefore, we selected literature with more than three citations as the high-frequency literature and selected the literature from 2013 to 2022 for content analysis.

TF addresses a wide range of issues, serves a variety of users, and is based on a variety of knowledge and information, using a variety of tools and methods to address a broad range of technology-related issues. Figure 4 illustrates the complex interactions between different contexts and agents in the TF system. In Figure 4, it can be seen that: technology opportunity identification [109] and technology trend and evolution analysis [110] are the two most concerning contexts for TF. The field of analysis covers an increasing range and has been extended to multiple fields for analysis [101]. The objects of analysis are mainly targeted at the technology [70] and industry [42] levels to provide more guided foresight. In terms of data sources, patents and publications still dominate. However, due to the rise of the concept of collaborative innovation, multi-stakeholder participation foresees the need to introduce multiple data sources [83], such as trademarks, online platforms and forums, and Wikipedia. With the massive growth of information resources, traditional expert opinions cannot meet the demand [2]. Additionally, more data mining, processing, and interpretation tools have emerged, and multiple methods are cross-used for more detailed and in-depth problem research [93].

#### 3.3.1. The Interactive Relationship in the Context of Technology Opportunity Identification

**Figure 5:**
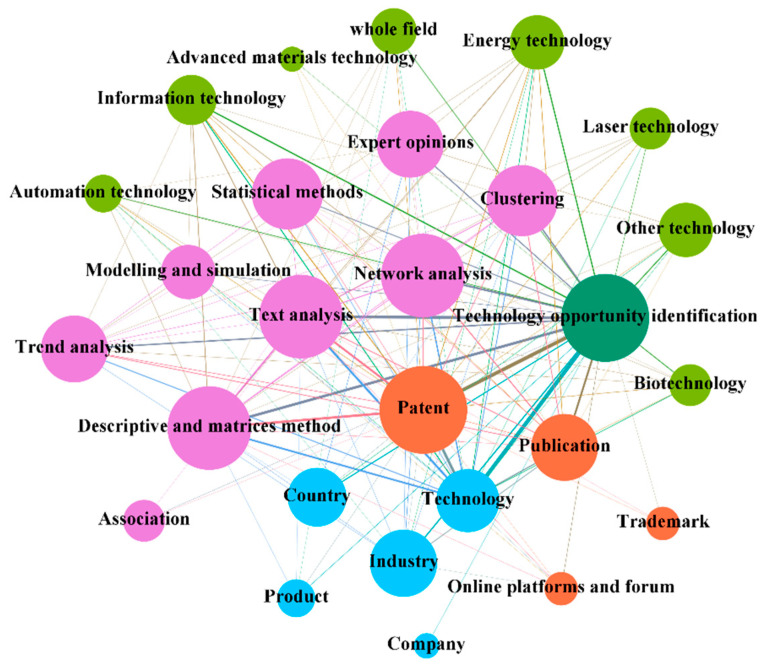
The figure shows the interactive relationships between the agents in the context of technology opportunity identification.

Technological opportunities are a set of possibilities for technological advances to improve product functionality or production [27]. Therefore, many researchers have been working on constructing paths to effectively identify and predict technological opportunities using agents in TF systems. The interactions between the agents involved in the context of technology opportunity recognition are shown in Figure 5.

1. Field of analysis. The development of technology has defined three successive societies: the industrial society, the information society, and the molecular society [6]. Research focusing on the identification of opportunities in automation technologies [86] has led to the recognition of the importance of systems analysis for the design of new systems. The fields of energy technology [97] and information technology [111] have been widely used to explore the technological advances that may arise during future development due to their great potential for creating social and economic value. Biotechnology and new material technologies [66] are currently in the gestational phase. In addition, opportunities conducted for a single field have the limitation of weak universality. Therefore, technology opportunity identification is gradually expanding to be conducted in multiple fields or even across the board [75].
2. Object of analysis. Opportunity identification revolves around technology, product, company, industry, and country levels; technology and industry level identification are more extensive. Among them, the technology [70], product [59], and company [79] level opportunities are more fine-grained and can provide more specific guidance for R&D subjects. The industry-level [32] and country-level [112] opportunity identification focus on the frontier technology opportunities at the field and industry levels. They serve as guides in the development of national innovation policies and the selection of technology benchmarks and directions by R&D entities.
3. Date source. Different levels of opportunity identification influence the choice of data sources; and patents, publications, trademarks, and online platforms and forums have been used for opportunity identification. Among them, technology-level opportunity identification is mostly conducted based on patent texts as important technology information carriers [64]. Meanwhile, technology and science connections can also be explored for mining with the help of patents and publications [2]. In contrast, industry-level opportunity identification is mostly based on multiple data sources, such as patents, publications, and online platforms and forums for multi-level opportunity identification of technologies, products, and markets [83]. In addition, patents and trademarks can be used to identify business opportunities at the product level [59].
4. Approach. The technology opportunity identification process is summarized in four steps: knowledge element extraction, knowledge element structuring, technology opportunity representation, and definition and technology evaluation. Firstly, text analysis can be used to extract knowledge elements from the data and achieve dimensionality reduction in the data [32]. Secondly, cluster analysis [64], association analysis [95], modeling and simulation [33], and network analysis [73] can be used to further explore the logical relationships between elements and structured representation of elements. Again, the representation and definition of technological opportunities can be achieved with the help of expert experience or descriptive and matrix analysis methods [47]. Finally, statistical methods [113] can also be used to test the robustness of the prediction results and to provide in-depth analysis and interpretation of them.

#### 3.3.2. The Interactive Relationship in the Context of Technology Assessment

**Figure 6:**
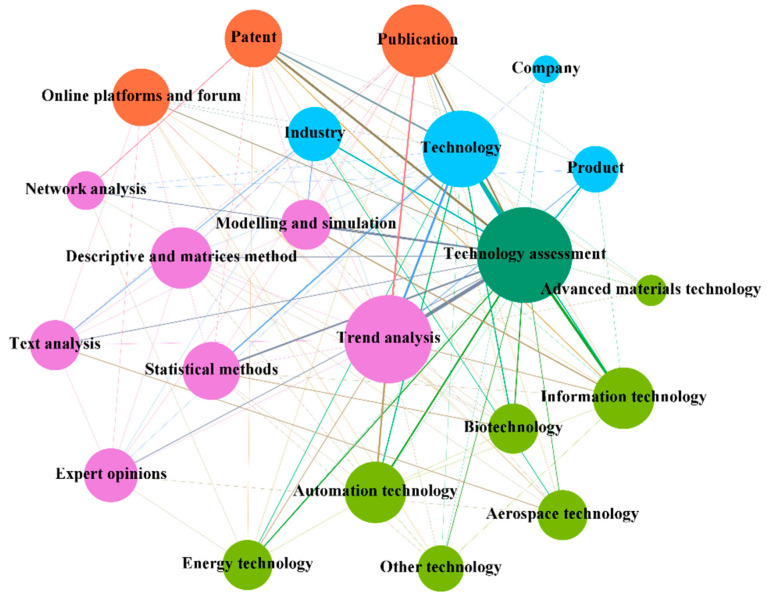
The figure shows the interactive relationships among the agents in the context of technology assessment.

Technology assessment aims to understand the potential social, economic, political, ethical, and other consequences of the introduction of new technologies or the diffusion of existing technologies, with a focus on unplanned and unanticipated [5]. As an established research area in the field of policymaking, researchers have systematically assessed the social and economic impacts of technology using the interactions between different agents. The interactions between the agents involved in the context of technology assessment are shown in Figure 6.

1. Field of analysis. Technology assessment can explain the favorable or unfavorable nature of consequences in multi-technology areas and guide R&D subjects to invest in socially beneficial components. By considering the dynamic nature and dynamics of technology impacts, more accurate technology impacts can be provided for the information technology field, which has a short innovation cycle [52]. Additionally, future-oriented experts and public opinions are collected to identify and understand the overall profile of unintended consequences of emerging technologies in the aerospace technology sector [51]. In addition, technology assessments were used to measure the maturity and life cycle of automation technologies [58], biotechnology [35], and energy technologies [37].
2. Object of analysis. Technology assessment revolves around the technology, product, company, and industry levels; the technology level is most prominently used. Technology and product-level assessments are mostly based on technology maturity to monitor changes in the speed and maturity state of technology development and to guide R&D entities in making strategic decisions [38]. Industry-level assessments focus on exploring the various implications of policy, protection, evaluation, and commercialization related to the adoption and deployment of new technologies [114].
3. Date source. Patents, publications, and online platforms and forums are used to enable technology assessment. Among them, patents [115], publications [52], and online platforms and forums [38] can be used in the process of technology life cycle analysis at the technology and product levels to measure the stage of technology and future trends. In addition, the collective wisdom of experts and public participation in online platforms and forums can also be used to identify unknown risks arising from future technologies [51] and to study the impact of the technology on the whole social system. Compared with those traditional data sources, web-based information has a high acceptance rate and is faster and more accurate.
4. Approach. Traditional patent-based [115] and publication-based informetric [36] approaches are at the forefront of research on maturity assessments and related tasks. They can also be combined with methods such as network analysis and expert opinion for finer-level technology lifecycle analysis [37]. However, due to the advantages possessed by online platforms and forums, text mining techniques are used to mine potential technology information and construct technology evaluation metrics using multi-perspective assessments [38]. This approach is a useful complement to traditional methods. In addition, modeling and simulation methods are used to explore the various implications of policy, protection, evaluation, and commercialization associated with big data and its applications [114].

#### 3.3.3. The Interactive Relationship in the Context of Technical Trend and Evolution Analysis

**Figure 7:**
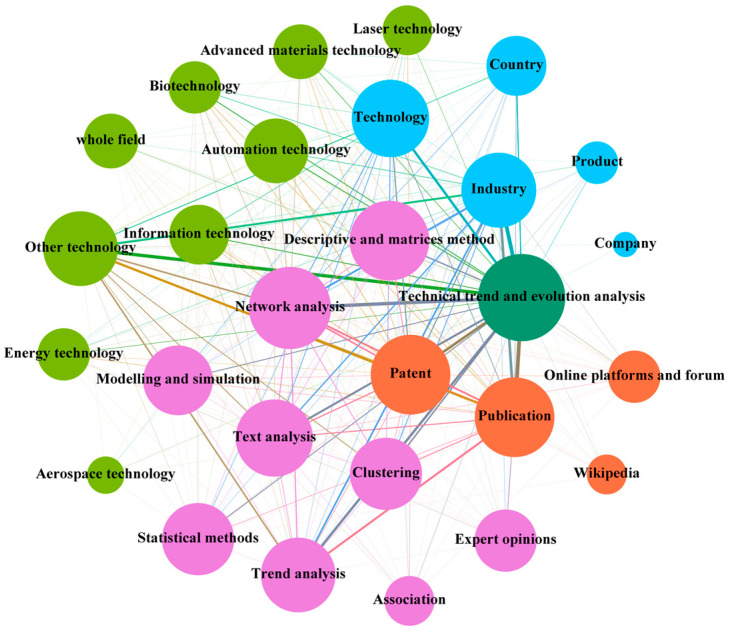
The figure shows the interactive relationships among the agents in the context of technical trends and evolution analysis.

As one of the most important drivers of economic development, effective mastery of technology trends and evolutionary processes is essential for developing better product and service capabilities [68]. Increasingly complex technological relationships have prompted researchers to view interacting and interdependent technologies as complex systems. Additionally, the complex interactions among agents are used to grasp technology trends and evolution. The interactions between the agents involved in the context of technical trend and evolution analysis are shown in Figure 7.

1. Field of analysis. Technology evolution pathways paths can be used to understand and analyze the development of technology topics in multiple fields. The fields of automation technology [93], laser technology [68], and new materials technology [65] are probed for technological evolution over a specific time to quickly identify research hotspots and gaps. It can also be used in multiple fields to demonstrate the complex evolutionary relationships between technologies, products, and markets [116]. In addition, diffusion and productivity multi-macroeconomic models are used to explore empirical patterns of heart attack survival gains in the biological field [117]. As in other contexts, the analysis of technology trends and evolution extends to multiple fields to explore the trajectory of future technologies [101].
2. Object of analysis. Technology trends and evolutionary analysis involve multiple levels of technology, products, companies, industries, and countries; the technology and industry levels are the most widely used. Technology-level studies mostly focus on understanding the process of technology transfer and exploring its specific forms of association [65]. Industry-level studies focus on exploring the linkages between different levels of the technology roadmap and measuring the dependencies between the elements of each level, based on which, trend analysis and strategy formulation can be carried out [42]. In addition, country-level studies can reveal significant research opportunities and help institutions and researchers around the world clarify potential research gaps [118].
3. Date source. Patents, publications, online platforms and forums, and Wikipedia are used for technology trends and evolution analysis. Publications contain textual information, citation information, and reliable information on technological developments [93]. When analyzing the development of a specific field, the textual information of publications can be used to reveal the development of the subject matter [81], but citation relationships cannot be ignored [119]. Patents can be used for technology diffusion analysis [8] and also for technology route mapping [42] to deeply analyze the correlation relationships between technologies. Online platforms and forums can be used as supplementary information to enrich the information contained in patents and publications to make the trend analysis more systematic [53].
4. Approach. Text analysis [120] and network analysis [44] have been used to mine textual information and citation relationships for patents and publications, respectively, and can also be used in combination to achieve finer-grained studies of technology [121]. In addition, bibliometrics can be used to explore, organize, and quantitatively analyze large volumes of scientific literature [122]. It is often used in conjunction with methods such as cluster analysis [123] and statistical methods [68] to reveal the current state of research in the field of study. Statistical methods can also provide tools to support the elucidation of spatial technology trajectories and reveal future developments [92].

#### 3.3.4. The Interactive Relationship in the Context of “Others”

**Figure 8:**
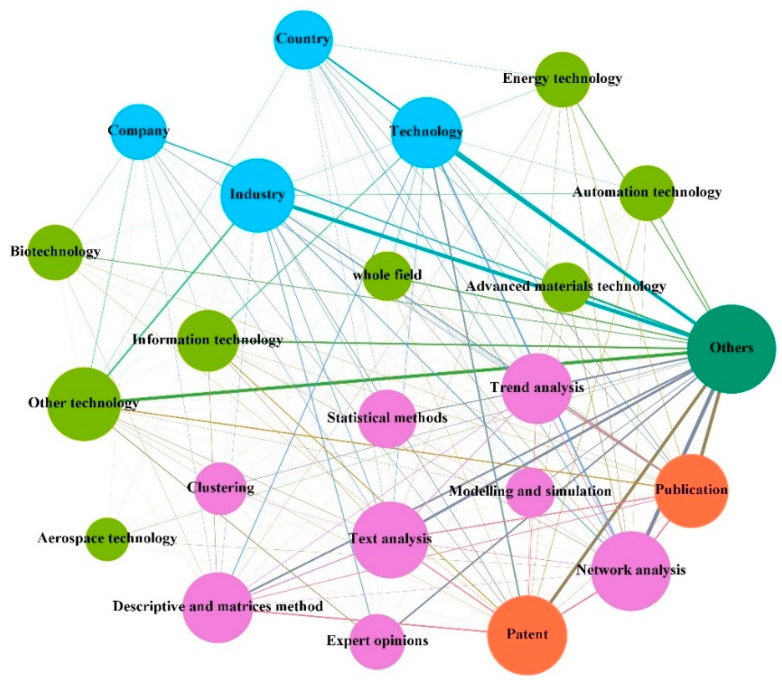
The figure shows the interactive relationships between the agents in the context of “others.”

The main research contents in this context are scattered and less concerning. However, as a part of the TF system, small changes will also cause major consequences in the system. As the complex system TF is open, changes in the feedback loop will change the overall behavior of the system. The interactions between the agents involved in the context of technical trend and evolution analysis are shown in Figure 8.

1. Field of analysis. The context involves applications in other fields, such as the environmental research field studying gaps and priorities for implementation decisions [124] or exploring extreme AI labor transfer scenarios [99]. TF is not limited to the development of the technology itself, but also requires estimates of the context of future developments and their possible effects, involving multiple fields, such as science, technology, economics, and politics. In addition, the fields of information technology [91], biotechnology [104], and energy technology [57] have also been explored in many ways to find future-oriented insights for their industries.
2. Object of analysis. The context involves the technology, firm, industry, and country levels, having a primary focus on the technology and industry levels. Technology-level studies have been conducted to explore whether major inventions are more technologically diverse and the existence of innovation patterns [125]. At the industry level, temporal factors are considered to extend scenario discovery, which has been used for decision support purposes [126]. Additionally, a firm-level competitive advantage can be explored around the management of emerging technologies and traditional firm capabilities [12]. Interdisciplinary collaboration at the national level is studied, as harmonious academic relationships help to strengthen scientific collaboration networks [45].
3. Date source. This context involves two types of data sources: publications and patents. Important technical information can be retrieved from IPC and text information based on patent data to determine the objectives of technology acquisition [46]. Patents can also be used as a source of information to analyze the breadth and depth of knowledge within companies, as data provide insight into their capabilities [56]. At the same time, the company’s patent portfolio and the evolution of the timeline are also important for formulating technical strategies for different competitors [74]. In addition, co-indigenous information contained in publications can be used to explore the evolution of institutional cooperation over time [127]. This is because co-authorship is one of the most specific and well-documented forms of scientific ensembles.
4. Approach. Author co-authorship networks have been constructed to explore patterns of scientific collaboration at the national, institutional, and individual levels [128]. Additionally, citation networks can be used to examine the network structure and knowledge spillover channels of firms [55]. Network analysis can also be used in conjunction with text analysis, trend analysis, statistical methods, and descriptive and matrix methods for node mining at the front end of the network and analysis at the back end. Expert opinion [54] serves as a guarantee of TF reliability throughout the process of TF. In addition, cluster analysis can explore the current state of R&D and capture future development signals and trends [46].

## 4. Discussion

The systematic and adaptive nature of TF indicates that it is a response to a changing environment and provides a mindset for meeting challenges [77]. However, despite calls from industry practitioners to better investigate the relationship between the context, content, and mindset of TF, such explicit mapping has not yet been presented. It allows for the alignment of TF behavior with the goals pursued. In this regard, we recognized the basic elements of the system and gained insight into the operation of the whole system with the help of the network based on the clarification of the system context and the subjects involved. Additionally, the results should help design and develop TF approaches in different contexts.

Firstly, the interaction relationship network can be used as a guiding model for TF activities. Given the complexity of the innovation process and competitive system, understanding the diverse and increasingly complex tools allows critical information to be obtained from multiple sources. In the case of different problem solving and goal pursuits, R&D subjects should first clarify the specific context and then select the corresponding agents in the network use TF effectively. In addition, R&D subjects can also match their resources and technical skills with the corresponding TF behavior to achieve incremental innovation. This information will help avoid dead ends and fatal surprises, and seize technology opportunities in a competitive marketplace.

Secondly, the interaction relationship network can be used as a reference model for TF activities. R&D subjects can expand and modify different agents according to the existing contexts and the association relations between agents to achieve more accurate TF contexts. This measure can be described as follows: (1) to define the context (technology opportunity identification); (2) to specify the technical field of analysis (information technology); (3) to select the level of analysis (technology); (4) to select the corresponding data source (patent); (5) to determine the method to realize technology forecasting (statistical method). Among them, various measures can be employed to improve the effectiveness and accuracy of TF, such as the integration of multiple source databases, the optimization of prediction methods, and a combination of them.

However, note that TF is a complex system in which adhesion occurs through interrelated cooperation between agents to achieve different tasks and goals, rather than a simple linear process. In addition, the system is constantly adapted to new environments as data collection, processing, and interpretation tools are continuously optimized.

## 5. Conclusions

Many studies have focused on the field of TF, but systematic studies that can further contribute to the outcomes of TF are yet to be explored. In this paper, future research trends in this field were identified based on a systematic analysis of TF (Table 4).

1. Refinement of the context focus on combining technologies and the broad social impacts they generate to provide concrete and effective decision guidance to R&D subjects. We note two issues in this category. Firstly, the result of TF determines the success or failure of prediction, yet existing studies mainly conduct TF from a methodological perspective, lacking a systematic evaluation of prediction results. Therefore, the output of the technology, along with the social impact, should be evaluated to make realistic decisions. On the other hand, the available R&D resources should be assessed to ensure that the prediction goals are achieved. Secondly, the different interactions between agents in different contexts mean that prediction methods are not universally applicable. Therefore, the synergistic mechanism between agents in different contexts needs to be explored in-depth in the future. Then, more context-specific prediction methods need to be developed and designed to provide realistic guidance.
2. Optimization and expansion of the analysis field emphasize the optimization study of existing technology fields and the expansion of new technology fields to expand the application scope and verify the universality of prediction methods. Two issues in this category were noted. Firstly, most of the existing studies have been conducted on the practical use of TF for a single field. However, the implementation of TF is a complex, systemic act that includes not only the systematic analysis of technology research directions and generic technologies, but also the study of the economic and social issues associated with them. Therefore, the universality of the forecasting method needs to be further verified in multiple fields. Secondly, TF can anticipate the direction and speed of technological changes, aiming for rational decision-making on technology. Therefore, TF in more technology areas should be conducted to effectively guide relevant practitioners to achieve decision-making and capital investment.
3. Extension of the analysis object highlighted multi-level analysis and multi-subject participation mechanisms aiming to enhance the impact of prediction results. TF at different levels has an important role in shaping technology to meet the development needs of society. TF is widely used at the technology level as technical support for strategic planning or opportunity identification. At the industry level, it can be used to generate visions to guide the future with the consensus of decision-makers and stakeholders. At the country level, it is important for measuring the logical relationship between technology and industry, which aims for competitive advantages and effective resource allocation. At the company and product levels, it can also be used as part of the collection and analysis of competitive intelligence and strategic planning for product development. The current study has gradually shifted to multi-level forecasting systems for countries, industries, and companies; and in the future, it could also involve multi-stakeholders in the activities of TF and integrate resources from multiple parties to enhance the impact of prediction results.
4. Convergence and diversification of the data source concern the integration of existing databases and the development of new databases to increase the credibility of prediction results. The credibility of knowledge and information materials can increase the credibility of TF results. In terms of its data sources, the existing TF studies show the characteristics of mainly patent and publication use, giving less consideration to online platforms and forums, trademarks, and Wikipedia information. Different types of data sources have different focuses. Data sources are high-quality sources of information on the latest research developments in technology. Publications are the main sources of information on technology research and applications. Trademarks involve applicant information that can be used as a supplementary resource for competitive intelligence analysis. Online platforms and forums and Wikipedia involve the views of other stakeholders, making available more comprehensive information. Therefore, the existing data sources can be integrated to obtain a more comprehensive information. In addition, other types of data information can be integrated to lay the data foundation for accurate and comprehensive TF activities.
5. Combination and optimization of the approach emphasize four aspects of research to improve the quality and accuracy of prediction results. We identified the following four research agendas. Firstly, with the increasing development of TF activities and the growing maturity of related methods, there is a gradual trend of using a blend of multiple methods, as using a single rule to predict is prone to deviation. Therefore, multiple forecasting methods can be used through complementary mutual authentication and organic integration to achieve all-round TF. Secondly, the traditional keyword-based series approach makes it difficult to systematically consider the ability to characterize topic feature words in the field, which may lead to understanding deviation, and then affect the accuracy of results. Therefore, it is important to improve the accuracy of results by using the SAO semantic mining method, technology-relationship-technology semantic analysis, and other methods to carry out semantic analysis and deeply analyze its internal relations. Thirdly, the traditional static analysis of TF does not consider the time factor, so it cannot reveal the emphasis of research and development overtime changes. Therefore, it is an important way to improve the reliability of TF results by considering the time factor and exploring the changing rules and behavior of R&D priorities. Finally, regarding the complex data characteristics and environment, big data analysis technology and intelligent analysis technology should be fully utilized to process massive information to further improve the reliability of TF.

Although the results of this paper are relevant to assisting relevant practitioners in understanding and developing TF, there are still some limitations. Firstly, we only selected the literature published in the top 50 journals in the field of TIM for review to dissect the system of TF. More data databases, such as news, reports, and economic data, could be included in the future to expand the scope of the study. In addition, we did not analyze the temporal evolution of the contexts and agents involved in the system. The temporal evolution process of the agents involved in different contexts can be further analyzed in the future to explore the trend development.

## Figures and Tables

**Table 1:** Four contexts in the system of TF. Content analysis of the literature corresponding to the above clustering topics was conducted to clarify contexts and major research contents in the system of TF.

Contexts	Cluster	Main Research Contents	Ref.
Technology opportunity identification	#0 & #2 & #6 & #8	To identify the emerging technologies	[27,47]
		To explore disruptive technologies	[29,48]
		To discover vacant technologies	[32,49]
Technology assessment	#1 & #5	To extract key technologies	[33,50]
		To deal with the entire system that analyses the effects and the causes	[35,51]
Technical trend and evolution analysis	#3 & #4 & #10 & #11 & #13 & #14	To assess the maturity and life cycle of technology	[37,52]
		To depict the evolvement of technology across a certain time span	[7,39]
		To show the inter-relationship between market, product, and technology	[42,53]
		To track the process of technology spreading through specific paths in society	[9,43]
Others	#7 & # 9 & #12	To trace the changing industrial competition and collaboration	[45,54]
		To explore the depth and breadth of knowledge and technological trajectories	[55,56]
		To define and develop the efficient decision support system	[57]

**Table 2:** The table shows the eight technology fields and the corresponding concrete fields involved in the system of TF.

Technology Field	Concrete Fields (Selected)	Ref.
Information technology	Information technology field; Information and communication technology field; Competitor intelligence; Human-computer interaction technology	[33,62,63,64]
Advanced materials technology	Nanowire; Semiconductor foundry industry field; Graphene; Solid lipid nanoparticles field	[50,55,65,66]
Energy technology	Liquid biofuel niche; Perovskite solar cell technology; Solar PV and wind power field; Dye-sensitized solar cell	[2,27,37,57]
Laser technology	Radio Frequency Identification field; Coherent light generators field; Thermal management technology of light-emitting diode field	[67,68,69]
Automation technology	Artificial intelligence research field; Computer numerical control machine tool; Machine-building industry; 3D printing technology	[9,29,46,70]
Aerospace technology	Fighter jets and commercial airplane field; Drone technology field; NASA Astrobiology Institute	[51,71,72]
Biotechnology	Malignant melanoma of the skin; Cognitive rehabilitation therapy; genetically modified crops; Alzheimer’s disease research	[35,44,73,74]
Other technology	Whole field; Retail industry; B2B market; Health insurance service firm	[12,41,75,76]

**Table 4:** The table describes the specific research directions of TF system in terms of contexts and agents.

Theme	Sub-Themes
Refinement of the context	Enhancing systematic assessment of the results of TF
	Developing more targeted technology forecasts
Optimization and expansion of the analysis field	Conducting multi-field and even field-wide technology forecasting
	Expanding into new technology applications
Extension of the analysis object	Conducting comprehensive multi-level analysis
	Upgrading the participation mechanism to guarantee the professionalism of TF
Convergence and diversification of the data source	Concerning the converged use of multi-source databases
	Expanding new databases
Combination and optimization of the approach	Shifting from a single prediction method to a combination of multiple methods
	Exploring semantic mining methods in focus
	Considering the time factor to achieve dynamic forecasting
	Introducing new intelligent analysis tools to improve the level of excavation

## Data Availability

Data were obtained from the Web of Science Core Library database and available at https://www.webofscience.com (accessed on 24 November 2021).
